# Contribution of Panton-Valentine Leukocidin in Community-Associated Methicillin-Resistant *Staphylococcus aureus* Pathogenesis

**DOI:** 10.1371/journal.pone.0003198

**Published:** 2008-09-12

**Authors:** Binh An Diep, Amy M. Palazzolo-Ballance, Pierre Tattevin, Li Basuino, Kevin R. Braughton, Adeline R. Whitney, Liang Chen, Barry N. Kreiswirth, Michael Otto, Frank R. DeLeo, Henry F. Chambers

**Affiliations:** 1 Division of Infectious Diseases, Department of Medicine, San Francisco General Hospital, University of California San Francisco, San Francisco, California, United States of America; 2 Laboratory of Human Bacterial Pathogenesis, Rocky Mountain Laboratories, National Institute of Allergy and Infectious Diseases, National Institutes of Health, Hamilton, Montana, United States of America; 3 Public Health Research Institute and the University of Medicine and Dentistry of New Jersey, Newark, New Jersey, United States of America; Columbia University, United States of America

## Abstract

Community-associated methicillin-resistant *Staphylococcus aureus* (CA-MRSA) strains typically carry genes encoding Panton-Valentine leukocidin (PVL). We used wild-type parental and isogenic PVL-deletion (Δ*pvl*) strains of USA300 (LAC and SF8300) and USA400 (MW2) to test whether PVL alters global gene regulatory networks and contributes to pathogenesis of bacteremia, a hallmark feature of invasive staphylococcal disease. Microarray and proteomic analyses revealed that PVL does not alter gene or protein expression, thereby demonstrating that any contribution of PVL to CA-MRSA pathogenesis is not mediated through interference of global gene regulatory networks. Inasmuch as a direct role for PVL in CA-MRSA pathogenesis remains to be determined, we developed a rabbit bacteremia model of CA-MRSA infection to evaluate the effects of PVL. Following experimental infection of rabbits, an animal species whose granulocytes are more sensitive to the effects of PVL compared with the mouse, we found a contribution of PVL to pathogenesis over the time course of bacteremia. At 24 and 48 hours post infection, PVL appears to play a modest, but measurable role in pathogenesis during the early stages of bacteremic seeding of the kidney, the target organ from which bacteria were not cleared. However, the early survival advantage of this USA300 strain conferred by PVL was lost by 72 hours post infection. These data are consistent with the clinical presentation of rapid-onset, fulminant infection that has been associated with PVL-positive CA-MRSA strains. Taken together, our data indicate a modest and transient positive effect of PVL in the acute phase of bacteremia, thereby providing evidence that PVL contributes to CA-MRSA pathogenesis.

## Introduction

The worldwide emergence of community-acquired methicillin resistant *Staphylococcus aureus* (CA-MRSA) strains has been linked to carriage of genes encoding Panton-Valentine leukocidin (PVL), a two-component leukolytic toxin [1]–[9]. The contribution of PVL to CA-MRSA pathogenesis remains controversial. No difference in virulence was detected when comparing two prevalent CA-MRSA strains, LAC (USA300 lineage) and MW2 (USA400 lineage), to their respective isogenic PVL knockout mutants in several mouse models, including subcutaneous abscess, sepsis and pneumonia models [10]–[13]. Data supporting a role for PVL in pathogenesis are derived from experiments in a mouse pneumonia model using laboratory strains of the NCTC8325 lineage lysogenized with a PVL-encoding bacteriophage [14]. Presence of PVL was associated with up-regulation of staphylococcal protein A (Spa) and other surface proteins and led the investigators to propose a model in which PVL interference with global regulatory networks culminated in overwhelming inflammation and necrosis of the murine lung [14]. Such profound effects on global gene expression raise the possibility that the experimental outcomes were due not to PVL, but were consequence of major genetic perturbations [9], perhaps due to pleiotropic mutations that occur with relatively high frequency in laboratory strains [15], [16].

The conflicting data from mouse infection models and the relative insensitivity of murine polymorphonuclear leukocytes (PMNs or granulocytes) to the leukolytic effect of PVL compared with human cells prompted us to assess the role of PVL in CA-MRSA pathogenesis in a rabbit model. Importantly, the sensitivity of rabbit PMNs to the leukolytic activity of PVL mirrors that of human PMNs [17], making the rabbit an excellent model species because PMNs are a primary cellular target of PVL and the principal component of host innate immune defense. Intravenous injection of purified PVL into rabbits results in transient granulocytopenia followed by marked granulocytosis, but is not lethal [18]. Here, we tested in prevalent CA-MRSA strains whether PVL regulates global gene networks and evaluated its contribution to pathogenesis of bacteremia, a hallmark feature of invasive *S. aureus* disease and the most prevalent clinical syndrome of invasive CA-MRSA disease in particular [19].

## Results

### PVL does not impact global gene regulation

Introduction of PVL into a laboratory strain of *S. aureus* was reported to alter global gene regulation, resulting in increased expression of surface adhesins such as staphylococcal protein A (Spa) [14]. To assess the potential regulatory effects of PVL in clinically relevant CA-MRSA strains, we performed experiments using contemporary CA-MRSA belonging to the two prevalent lineages, USA300 and USA400 [3]–[6], [20].

**Figure 1:**
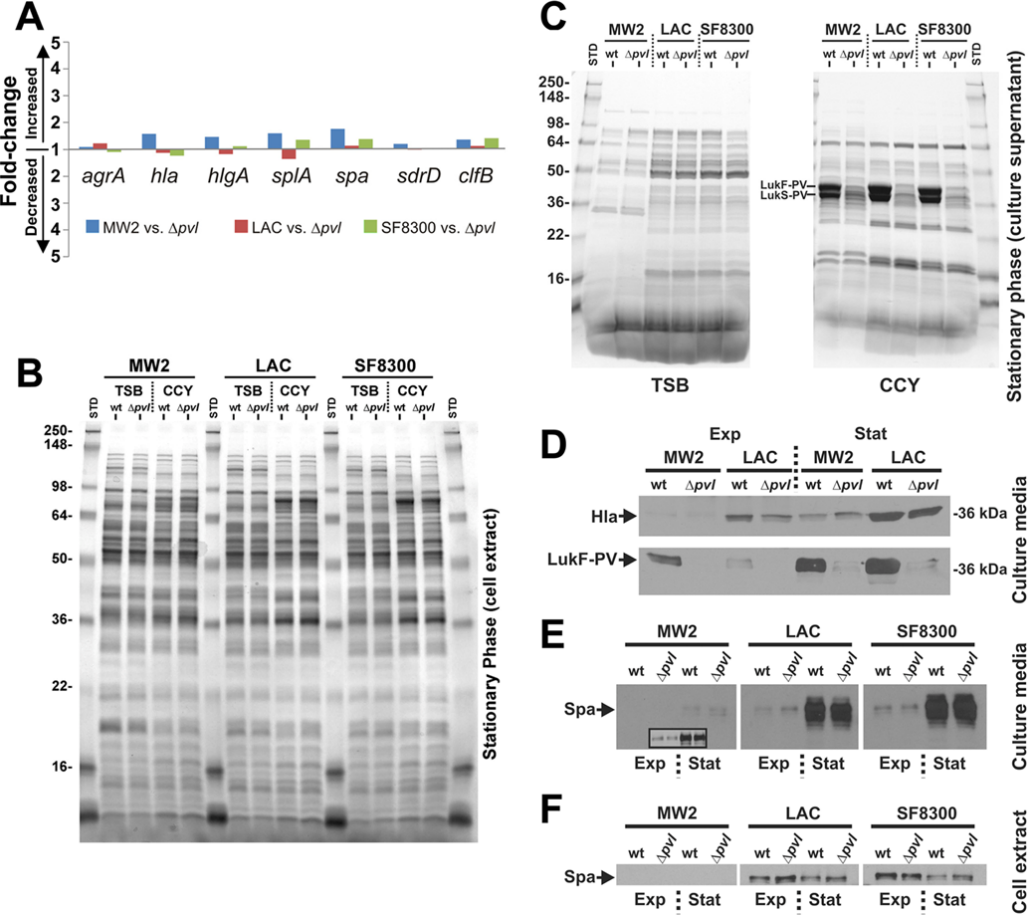
PVL does not alter global gene and protein expression profiles. Clinical strains of USA300 (LAC and SF8300) and USA400 (MW2) and their respective isogenic Δ*pvl* mutant strains were cultured to mid-exponential or stationary phases of growth in TSB or CCY media. (A) TaqMan real-time RT-PCR for comparison of fold changes in transcript levels of selected Agr-regulated genes in wild type and Δ*pvl* mutant strains. See also Table 2 for additional data derived from *in vitro* growth to exponential phase and stationary phase in CCY or TSB media. *agrA*, accessory global regulator; *hla*, alpha-toxin; *hlgA*, gamma-haemolysin component A; *splA*, serine protease; *spa*, protein A; *sdrD*, serine aspartate repeat protein; *clfB*, clumping factor B. (B) Cell extracts separated by 12% SDS-PAGE (Protean II gel, Bio-Rad) using cultures grown to stationary phase. (C) Culture supernatants, prepared from growth in TSB or CCY media, were separated by gradient 10-20% SDS-PAGE. PVL subunits were identified by automated-direct infusion tandem mass spectrometry [32]. (D, E, F) Western immunoblot analysis of supernatants and cell extracts from cultures grown to stationary or mid-exponential phase. Proteins were detected with rabbit polyclonal antibodies specific for LukF-PV, Hla (α-toxin), or Spa. The immunoblots in panel E were exposed on the same film for equal times or using a longer exposure for MW2 (inset), which produced less Spa. Protein samples for SDS-PAGE presented in panels B and C were prepared in a manner identical to those shown in panels E and F.

To examine the effect of PVL on global gene expression, we conducted transcriptional profiling of wild-type parental and isogenic Δ*pvl* mutant USA300 (SF8300–SF8300Δ*pvl*) and USA400 (MW2–MW2Δ*pvl*) strain pairs. Total RNA was isolated after *in vitro* culture to exponential-or stationary phase of growth under conditions known to induce over-expression of PVL. Contrary to results reported by Labandeira-Rey et al. [14], only 1 of 3961 *S. aureus* microarray probesets met standard criteria for differentially-expressed genes (>2-fold change in transcript levels, a ratio of wild-type versus Δ*pvl* mutant strain and *P*<0.05 [21], [22]) using the SF8300–SF8300Δ*pvl* strain pair comparison. The single differentially-expressed gene, *spectinomycin adenylyl-transferase* (*spc*), was used for allelic replacement of *pvl* and thus present in the SF8300Δ*pvl* mutant strain but not in the wild-type parental strain (Table 1). Only 5 of 2632 MW2 probe sets were differentially expressed in the MW2–MW2Δ*pvl* strain pair comparison, and of these, 2 were specific for *lukS-PV* and *lukF-PV* found only in the MW2 parent strain (Table 1). Using TaqMan real-time reverse transcriptase-PCR, we confirmed that expression of PVL does not alter transcripts encoding accessory gene regulator (AgrA) and Agr-regulated virulence factors such as protein A (Spa), α-toxin (Hla), β-hemolysin (HlgABC), serine aspartate repeat protein (SdrD), serine protease (SplA), or clumping factor B (ClfB) (Figure 1*A* and Table 2).

**Table 1:** PVL does not alter global transcriptional profiles of USA300 and USA400.*

Strains	Media	Growth Phase	Differentially Expressed Gene€	
			No. of Genes	Gene Identification¥
SF8300 vs. SF8300Δ*pvl*	TSB	Exponential	1	*spc*
SF8300 vs. SF8300Δ*pvl*	TSB	Stationary	1	*spc*
SF8300 vs. SF8300Δ*pvl*	CCY	Exponential	0	–
SF8300 vs. SF8300Δ*pvl*	CCY	Stationary	1	*spc*
MW2 vs. MW2Δ*pvl*	TSB	Exponential	0	–
MW2 vs. MW2Δ*pvl*	TSB	Stationary	2	*lukF-PV* and *lukS-PV*
MW2 vs. MW2Δ*pvl*	CCY	Exponential	0	–
MW2 vs. MW2Δ*pvl*	CCY	Stationary	5	*lukF-PV*, *lukS-PV*, *set*, *lukE*, *plc*

€ Transcriptome analyses of SF8300 vs. SF8300Δ*pvl* mutant strains were performed using custom Affymetrix GeneChips (RMLChip1) containing 3961 probe sets from eight different *S. aureus* strains (COL, EMRSA16, MSSA476, RF122, TSS, 8325, Mu50, and N315); and MW2 vs. MW2Δ*pvl* using custom Affymetrix GeneChips (RMLChip3) with 99.3% coverage of genes from MW2 (2613 probe sets of 2632 ORFs; the remaining 0.7% are represented by identical probe sets from other staphylococci). Note that RMLChip1 does not contain probesets for *lukS-PV* and *lukF-PV*, which was subsequently assayed by TaqMan real-time RT-PCR (Table 2). Table displays only probesets that met standard criteria required for differentially-expressed genes (>2-fold change in transcript levels in the wild-type vs. Δ*pvl* strain, and *P*<0.05 using a unpaired Student's *t* test).

¥ *spc*, encoding spectinomycin adenylyltransferase (detected only in SF8300Δ*pvl* by RMLChip1; probe set absent in RMLChip3); *lukF-PV* and *lukS-PV*, encoding PVL (detected only in MW2 by RMLChip3; probe sets absent from RMLChip1); *set*, encoding an enterotoxin homolog (*MW0052*), 2.4 fold-change; *lukE* (*MW1768*), encoding leukocidin E, 2.1 fold-change; and *plc* (*MW0070*), encoding 1-phosphatidyl-inositol phosphodiesterase precursor, 2.6 fold-change.

**Table 2:** TaqMan real-time RT-PCR analysis reveals that PVL does not alter *agr*-regulated transcripts in USA300 and USA400 clinical strains.±

Strain	Growth Phase	*agrA*	*spa*	*clfB*	*sdrD*	*hla*	*hlgA*	*splA*	*lukF-PV*
		mean fold-change in gene transcripts for wt vs. Δ*pvl*							
For cells grown in TSB									
LAC vs. LACΔ*pvl*	Exponential	−1.25	−1.39	1.15	−1.36	−1.43	−1.25	−1.10	20769.59*
SF8300 vs. SF8300Δ*pvl*	Exponential	−1.31	−1.20	−1.31	−1.49	−1.32	−1.39	−1.28	48376.47*
MW2 vs. MW2Δ*pvl*	Exponential	−1.00	−1.24	1.40	−1.18*	−1.12	−1.46	−1.11	9092.34*
LAC vs. LACΔ*pvl*	Stationary	1.42	1.36	1.47*	1.27*	1.51	−1.06	−1.09	60925.20*
SF8300 vs. SF8300Δ*pvl*	Stationary	1.16	1.40	1.35	−1.04	1.58	1.22	1.82	126494.09*
MW2 vs. MW2Δ*pvl*	Stationary	1.37	1.52	1.63	1.39	1.44	1.95	1.67	16747.59*
For cells grown in CCY									
LAC vs. LACΔ*pvl*	Exponential	−1.23	−1.74	−1.57	−1.62	1.13	−1.64	−1.43	2133.83*
SF8300 vs. SF8300Δ*pvl*	Exponential	−1.10	1.27*^a^*	−1.15	−1.13	−1.20	−1.24*	1.05	4008.34*
MW2 vs. MW2Δ*pvl*	Exponential	−1.02	2.83	1.57	1.20	−1.76	−1.09	−1.39	7972.35*
LAC vs. LACΔ*pvl*	Stationary	1.22	1.13	1.12	−1.00	−1.12	−1.17	−1.35	392409.30*
SF8300 vs. SF8300Δ*pvl*	Stationary	−1.09	1.38	1.41	1.03	−1.23	1.11	1.35	781001.79*
MW2 vs. MW2Δ*pvl*	Stationary	1.08	1.76	1.36	1.20	1.57	1.46	1.59	146821.31*

± TaqMan real-time RT-PCR was performed as described in Methods. Results are expressed as the mean fold-change of 3–5 experiments (exponential growth, Exp.) or 4–7 experiments (stationary phase of growth, Stat.) with one exception*^a^* (one of the TaqMan reactions failed, n = 2). The relative expression level of each transcript (dCT) was compared in parent vs. Δ*pvl* strains using a paired Student's *t*-test (^*^ *P*<0.05 versus Δ*pvl*). Except for *lukF-PV*, none of the transcripts in any of the strains met standard criteria required for differentially expressed genes (>2-fold change in the wild-type vs. Δ*pvl* mutant strain and *P*<0.05).

*agrA*, accessory global regulator; *hla*, alpha-toxin; *hlgA*, gamma-haemolysin component A; *splA*, serine protease; *spa*, protein A; *sdrD*, serine aspartate repeat protein; *clfB*, clumping factor B; and *lukF-PV*, Panton-Valentine leukocidin component F.

Consistent with the microarray and TaqMan data, protein profiles of cell extracts and culture supernatants were virtually identical between each of the wild-type parental and Δ*pvl* isogenic mutant strains cultured with the same *in vitro* growth conditions (figure 1*B and 1C*). Although there was over-expression of PVL in supernatants of LAC, SF8300 and MW2 wild-type strains (figure 1*C*), the toxin did not modulate production of other virulence factors, including α-toxin and protein A (figure 1*D*–*F*). Protein A is a well-characterized proinflammatory factor mediating the development of disease in the lung [23]. However, we found that PVL does not modulate expression of protein A in CA-MRSA clinical strains, in marked contrast to what was reported by Labandeira-Rey et al. using laboratory strains of *S. aureus* [14]. Our results formally rule out the possibility that PVL contributes to CA-MRSA virulence by altering global gene and/or protein regulatory networks of *S. aureus*.

### Co-infection experiments in a rabbit model of bacteremia

As PVL has no effect on global gene and protein expression in prevalent CA-MRSA strains, a potential direct effect of PVL to CA-MRSA pathogenesis was examined. We used a rabbit model of bacteremia to compare wild-type parental and isogenic Δ*pvl* mutant strain pairs using a competition design. Rabbits were co-infected with a mixture of parent and Δ*pvl* mutant strains in an approximate 1∶1 ratio. The Δ*pvl* mutant strains contained a spectinomycin resistance cassette (*spc*) used to replace *pvl* genes, which allowed for the enumeration of the Δ*pvl* mutant (Spc-resistant) and parent strain (Spc-sensitive). Normalized ratios of the parent to Δ*pvl* mutant, representing competition indexes, were determined in organs harvested from rabbits that succumbed to infection or moribund rabbits with end stage bacteremia, which were euthanized between 2 and 7 days post infection (Table 3). The competition indexes in the rabbit lung, spleen, kidney and blood did not differ significantly from the null effect value of 0 for the SF8300–SF8300Δ*pvl* (n = 17), LAC–LACΔ*pvl* (n = 28) and MW2–MW2Δ*pvl* (n = 25) isogenic strain pairs, indicating no contribution of PVL to bacterial colonization and persistence at the end stages of bacteremia in the competition model.

**Table 3:** Co-infection experiments with USA300 or USA400 parental or isogenic Δ*pvl* mutant strains assayed at end stages of bacteremia.

	USA300		USA400
	SF8300 vs. SF8300Δ*pvl*	LAC vs. LACΔ*pvl*	MW2 vs. MW2Δ*pvl*
no. of rabbits	17	28	25
mean (±sd) inoculum (log_10_CFU)	7.42±0.49	7.80±0.31	7.45±0.25
mean (±sd) inoculum wt:Δ*pvl* ratio^1^	0.81±0.05	1.09±0.27	0.92±0.05
mean (±sd) survival in days	3.9±1.3	3.9±1.2	3.2±1.3
mean (±sd) bacterial density			
lung, log_10_(CFU/g)	2.77±1.39	3.31±1.46	3.05±1.84
spleen, log_10_(CFU/g)	2.64±1.51	2.97±1.35	2.66±1.25
kidney, log_10_(CFU/g)	3.70±1.83	4.95±1.69	4.41±1.97
blood, log_10_(CFU/ml)	1.55±1.29	1.14±1.15	1.33±1.00
competition index (95% confidence interval) 2			
Lung	−0.35 (−1.12–0.41)	0.30 (−0.14–0.74)	0.07 (−0.16–0.30)
Spleen	−0.03 (−0.75–0.69)	0.18 (−0.18–0.54)	0.12 (−0.03–0.27)
Kidney	−0.20 (−1.10–0.70)	0.66 (−0.01–1.33)	0.43 (−0.24–1.09)
Blood	−0.35 (−1.02–0.32)	−0.13 (−0.47–0.21)	0.21 (−0.16–0.58)

1 Competition assays were used to compare three wild type-Δ*pvl* mutant pairs: LAC-LACΔ*pvl* (n = 28), SF8300-SF8300Δ*pvl* (n = 17), and MW2-MW2Δ*pvl* (n = 25), where n is the total number of animals used in each experiment. A 1∶1 mixture containing approximately 3×10^7^ CFUs of wild type parent and 3×10^7^ CFUs of isogenic Δ*pvl* mutant were used to co-infect New Zealand white rabbits via the marginal ear vein. Mean bacterial densities comprising of both wild type and Δ*pvl* mutant from vital organs and blood are shown. The competition index (CI), which is the logarithm (log_10_) of the output ratios of parent and isogenic mutant after correction for variations in input ratios, are shown. A positive CI value indicates enhanced tissue infectivity of the parent, whereas a negative CI value indicates enhanced tissue infectivity of the mutant; a CI = 0 is the no-effect value.

2 The null hypothesis (CI = 0) that there was no difference in bacterial densities between parent and isogenic Δ*pvl* mutant in rabbit vital organs was tested using a paired Student's *t* test. All two-tailed *P* values were not statistically significant (*P*>0.05).

### Contribution of PVL to pathogenesis in a single-strain rabbit bacteremia model

Although PVL did not impact CA-MRSA pathogenesis in a competition bacteremia model, it is possible that secreted toxin from the parent produced a bystander effect that protected mutant cells. We assessed the potential contribution of PVL over the time course of bacteremia in the rabbit model in which either the parental strain SF8300 or the isogenic mutant SF8300Δ*pvl* were used to inoculate individual rabbits. Bacterial densities in vital organs determined at 24, 48 and 72 hours post infection (Table 4). In this model, bacterial densities decreased over time in the lung and spleen (linear test for trend, *P*<0.05), but increased in the kidney (linear test for trend, *P*<0.05), indicating that the kidney is a target organ that supports bacterial growth. At 24 and 48 hours post infection, significantly more SF8300 than SF8300Δ*pvl* were recovered from the kidney, but not from lung or spleen (*P*<0.05). In contrast, at 72 hours post infection, there was no significant difference in bacterial densities between SF8300 and SF8300Δ*pvl* isolated from kidney (Table 4). This result is in part explained by the rapid growth of 2.5 logCFU of SF8300Δ*pvl* in the kidney between 48 and 72 hours post infection (*P*<0.001), whereas this did not occur for the SF8300 parental strain (*P* = 0.63). The lack of difference in bacterial densities at 72 h post infection correlated with the end stages of disease in the bacteremia model, as rabbits infected with either SF8300 or SF8300Δ*pvl* had lost >15% of the baseline weight and some also exhibited other moribund conditions (Table 4). Moreover, there were no notable differences in gross pathology of kidneys between wild-type and Δ*pvl* mutant strains (data not shown). These results are consistent with a null effect of PVL at the end stages of bacteremia in the co-infection studies in which rabbits had a mean survival time of 3.9 days (Table 3).

**Table 4:** Time-course single-strain infection experiments with a USA300 parental or isogenic Δ*pvl* mutant strains.^1^

	SF8300wt	SF8300Δ*pvl*	*P* value
	log_10_CFU/g±standard deviations		
24 h post infection	n = 19	n = 19	
Lung	3.41±0.57	3.19±0.38	0.179
Spleen	3.62±0.57	3.44±0.38	0.27
Kidney	4.09±1.92	2.63±1.76	**0.020**
48 h post infection	n = 19	n = 18	
Lung	2.88±0.56	2.79±0.65	0.65
Spleen	2.72±0.90	2.64±0.82	0.78
Kidney	4.48±1.49	3.20±1.91	**0.030**
72 h post infection	n = 12	n = 12	
Lung	2.70±1.40	2.68±1.18	0.96
Spleen	2.70±1.34	3.03±0.78	0.47
Kidney	4.75±1.55	5.50±0.79	0.151
Blood			
24 h post infection	1.28±0.93	0.96±0.13	0.077
48 h post infection	0.68±0.16	0.50±0.13	0.37
72 h post infection	0.71±1.26	1.15±1.04	0.36

1 Rabbits were euthanized and log_10_CFU per gram of lung, spleen, and kidney were determined at 24, 48 and 72 hours post infection. It was not possible to conduct an experimental group at 96 hours post infection because rabbits loss >15% of the baseline weight by 72 hrs post infection, which is a moribund condition stipulated by UCSF animal use committee for euthanization. Two-sided *P* values by unpaired Student's *t* test are reported.

## Discussion

A PubMed search for articles on PVL published in 2002–2007 identified more than 300 articles, suggesting an association between PVL and CA-MRSA disease. Although compelling, epidemiological data alone are insufficient to establish whether PVL directly contributes to widespread dissemination of CA-MRSA strains or severity of infection [24], [25]. As bacteremia accounts for approximately 65% of invasive CA-MRSA disease [19], we used a rabbit model of bacteremia to study the role of PVL in CA-MRSA pathogenesis. Herein, we discovered a transient positive effect of PVL-mediated CA-MRSA pathogenesis in a rabbit bacteremia model. PVL appears to play a modest, but measurable role in pathogenesis during the early stages of bacteremic seeding of the kidney, the target organ from which bacteria were not cleared, as evidence by an increasing bacterial load over time (Table 4). During acute infection, it is possible that PVL-mediated lysis of incoming PMNs enabled better colonization and/or early survival of the parental USA300 strain. However, the early survival advantage conferred by PVL was lost by 72 hours post infection. Although it is unclear why there was no difference in bacterial densities in the kidney between parental wild-type and Δ*pvl* mutant strains at the end stage of bacteremia, it is worth noting that PVL has been shown to prime the host innate immune system [26]–[28] and this could have resulted in enhanced clearance of parental USA300 strain. For example, sublytic concentrations of PVL, orders of magnitude lower than required for granulocyte lysis, induce release of interleukin-8, leukotriene B_4_, and reactive oxygen species by PMNs, which contribute to innate host defense against bacteria [26]–[28]. In contrast to the parental USA300 wild-type strain, the Δ*pvl* isogenic mutant may not activate the immune response in this manner, allowing it to achieve significant growth in the kidney in the end stages of bacteremia and eventually compensate for any early survival advantage conferred by PVL. Alternatively, in established infection once a certain bacterial density has been achieved, PVL may play only a minimal role in maintaining infection.

Our study demonstrated clearly that there is no difference in transcriptome and/or proteome profiles between prevalent CA-MRSA wild-type strains and their isogenic Δ*pvl* mutants, indicating that a PVL effect must be direct and not mediated by interference with global regulatory networks (Figure 1 and Table 1). This stands in marked contrast to the regulatory role of PVL reported by Labandeira-Rey et al. [14] using a laboratory *S. aureus* strain lysogenized with a PVL-encoding phage [14]. While the reasons underlying these differences are under investigation , the absence of global gene regulatory effects of PVL in prevalent CA-MRSA strains may limit the practical applicability of the Labandeira-Rey et al. investigation [14]. Bubeck Wardenburg et al. [11], [12] found no contribution of PVL to pathogenesis in C57BL6 and BALB/c mouse pneumonia models using wild-type CA-MRSA and isogenic Δ*pvl* mutant strains. The differences in infection outcomes between the two studies could be related to differential levels of staphylococcal protein A (Spa), which was dramatically increased in the PVL-lysogenized laboratory strains [14], but remains unaltered in PVL-harboring CA-MRSA strains (Figure 1). Protein A has well-known inflammatory effects in murine lungs and ligates tumor necrosis factor receptor-1 (TNFR1) on airway epithelial cells [23].

In sum, we propose a model in which PVL exhibits a transient contribution to CA-MRSA pathogenesis in rabbit bacteremia model. Enhanced production of PVL in the early, acute phase of infection could contribute to CA-MRSA pathogenesis. This is consistent with the clinical presentation of rapid-onset, overwhelming infection that has been associated with invasive CA-MRSA disease in humans [29]. It is unclear why the effect is transient. Perhaps sublytic production of PVL in the end stages of infection could result in priming of the innate immune response that limits bacterial survival. Alternatively, once a threshold of organisms is achieved in a target organ, even if there is somewhat of a delay in getting to that threshold, factors other than PVL may be important in maintaining persistent infection.

## Materials and Methods

### Bacterial strains and culture

Clinical strains LAC and SF8300 are pulsed-field gel electrophoresis (PFGE) type USA300-0114, which have been implicated in epidemiologically unassociated outbreaks in the United States [20], [30]. MW2 is PFGE type USA400, the prototype CA-MRSA strain type endemic in the U.S. Midwest [3]. The isogenic PVL knockout (Δ*pvl*) strains, LACΔ*pvl* and MW2Δ*pvl*, have been described previously [10]. SF8300Δ*pvl* was constructed as described for LACΔ*pvl* and MW2Δ*pvl*, in which a spectinomycin resistance cassette replaced both the *lukS-PV* and *lukF-PV* genes. Bacterial strains were cultured in tryptic soy broth containing 0.25% D-glucose (TSB, Becton, Dickenson, and Company), CCY medium (3% [wt/vol] yeast extract, 2% Bacto-Casamino acids, 2.3% sodium pyruvate, 0.63% Na_2_HPO_4_, and 0.041% KH_2_PO_4_ [pH 6.7]), or YCP medium (3% [wt/vol] yeast extract, 2% Bacto-Casamino acids, 2% sodium pyruvate, 0.25% Na_2_HPO_4_, and 0.042% KH_2_PO_4_ [pH 7.0]). Overnight cultures were diluted 1∶200 and incubated at 37°C with shaking (250 RPM). Unless specified, bacteria were cultured to mid-exponential (TSB, OD_600_ = 0.75; CCY and YCP, OD_600_ = 1.0) or stationary (TSB, CCY, and YCP, OD_600_ = 2.0) phases of growth.

### Microarray experiments

SF8300, SF8300Δ*pvl*, MW2, and MW2Δ*pvl* were cultured to exponential (OD_600_ = 1.0) and stationary (OD_600_ = 2.0) phases of growth in TSB or CCY medium at which time bacteria were lysed using 700 µl of RLT buffer (Qiagen, Valencia, CA) and the lysate was homogenized using an FP120 FastPrep system (Qbiogene, Carlsbad, CA). Total RNA was isolated with RNeasy kits (Qiagen). Contaminating DNA was removed using DNase (on column DNase treatment,Qiagen; off column DNase treatment, Turbo DNase). Fragmented and biotin-dUTP-labelled cDNA was generated from purified RNA as described by the Affymetrix Target Preparation protocol (www.affymetrix.com/support/downloads/ manuals/expression_s3 _manual.pdf). To synthesize cDNA, random primers at 25 ng/ml (Invitrogen), 10 mM DTT, 0.5 mM dNTPs, 0.5 U/µl SUPERase·In (Ambion) and 25 U/µl SuperScript II (Invitrogen) were added to ∼20 µg RNA in 1X first-strand reaction buffer. The remaining RNA was hydrolyzed by adding 1 *N* NaOH at 65°C for 30 min after which 1 *N* HCl was added to neutralize the reaction. cDNA purification was performed using a QIAquick PCR purification kit (Qiagen) with nuclease-free water substituted for the elution buffer. cDNA (∼5 µg/sample) was fragmented using 0.6 U of DNase I (GE Healthcare) per mg of cDNA in One-Phor-All Buffer (Amersham Biosciences). Labeling of the 3′termini of the fragmentation products was completed as described in the protocol using 7.5 mM GeneChip DNA Labeling Reagent (Affymetrix), terminal deoxynucleotidyl transferase (Promega) in 5X reaction buffer. The reaction was terminated using 0.5 M EDTA. Biotinylated *S. aureus* cDNA from strains SF8300 and SF8300Δ*pvl* was hybridized to custom Affymetrix GeneChips (RMLChip1) containing 3961 probe sets from eight different *S. aureus* strains (COL, EMRSA16, MSSA476, RF122, TSS, 8325, Mu50, and N315). Biotinylated *S. aureus* cDNA from strains MW2 and MW2Δ*pvl* was hybridized to custom Affymetrix GeneChips (RMLChip3) with 99.3% coverage of genes from MW2 (2613 probe sets of 2632 ORFs; the remaining 0.7% are represented by identical probe sets from other staphylococci). GeneChips were scanned according to standard GeneChip protocols (Affymetrix). Precise details for Affymetrix hybridization and scanning protocols can be found at the above internet address. Each experiment was replicated 3 times. Affymetrix GeneChip Operating Software (GCOS v1.4, http://www.affymetrix.com) was used to perform the preliminary analysis of custom chips at the probe-set level. All *.*cel* files, representing individual biological replicates, were scaled to a trimmed mean of 500 using a scale mask consisting of only the *S. aureus* probe-sets to produce the *.*chp* files. A pivot Table with all samples was created, including calls, call *p*-value and signal intensities for each gene. The pivot Table was imported into GeneSpring GX 7.3 (Agilent), and hierarchical clustering (condition tree) using a Pearson correlation similarity measure with average linkage was used to determine similarity of biological replicates (data not shown). The pivot Table was also imported into Partek software (Partek Inc. St. Louis, MO) to produce a Principle Component Analysis plot as a secondary check on similarity of biological replicates (data not shown). After data had passed these preliminary statistical tests, biological replicates were combined into a custom worksheet (Microsoft Excel 2003, Microsoft Corporation) used to correlate replicates of all test conditions and controls. Quality filters based upon combined calls and signal intensities (test and/or control signal intensity was required to be greater than the average background signal intensity of 27) were used in the worksheet to further evaluate individual gene comparisons. Present and Marginal calls were treated as the same. Absent calls were negatively weighted for the filters and dropped completely from further calculations. All individual genes passing the above filters and combined from all usable replicates have the ratios of test (wild-type strain)/control (mutant strain) reported with associated probability computed by a paired Student's *t*-test. Significance Analysis of Microarrays (SAM) was also performed using the Excel sheet with a column added containing the results. *P*-values obtained from ANOVA (Partek) were filtered using the False Discovery Rate (FDR). Gene lists were generated with emphasis placed upon the quality and statistical filters mentioned above. To be included in the final gene list, in addition to the above criteria, gene expression must have been changed at least 2-fold. Microarray data are posted on the Gene Expression Omnibus (GEO, www.ncbi.nlm.nih.gov/geo/, platform accession numbers GPL2129 for RMLChip1 data (SF8300 strains) and GPL4692 for RMLChip3 data (MW2 strains), series accession number GSE8677).

### TaqMan real-time reverse transcriptase-PCR

TaqMan real-time RT-PCR analysis of 3–7 separate experiments (each assayed in triplicate) was performed with an ABI 7500 thermocycler (Applied Biosystems) using RNA samples prepared as described for the microarray experiments. Relative quantification of *S. aureus* genes was determined by the change in expression of target transcripts relative to the endogenous control gene, *gyrB*. Data were subsequently expressed as fold-change in wild-type transcript levels compared to the isogenic *lukS/F-PV* mutant strains (Δ*pvl* strains set at 1.0, baseline). *lukS/F-PV* transcripts were undetecTable in the Δ*pvl* strains.

### Analysis of *S. aureus* protein profiles

*S. aureus* were cultured to mid-exponential (TSB, OD_600_ = 0.75; CCY and YCP, OD_600_ = 1.0) or stationary (TSB, CCY, and YCP, OD_600_ = 2.0) phases of growth as described above. At the desired phase of growth, 10 ml of culture was centrifuged at 2800×g for 10 min at 4°C. Supernatant (5–7 ml) was collected and concentrated to 2X using Centriplus centrifugal filters with 3,000 MW cut-off membranes (Millipore, Bedford MA). Cell pellets were washed in 1 ml of cold Dulbecco's phosphate-buffered saline (DPBS) and resuspended in 600 µL of cold DPBS. Sample was loaded into a pre-chilled FastPROTEIN BLUE tube (MP Biomedical, Solon, OH) and *S. aureus* were disrupted/homogenized with a FP120 FastPrep Instrument (Qbiogene) set at speed 6.0 for 20 sec. Samples were immediately returned to ice. After homogenization, tubes were centrifuged at 10,000×g for 1 min at 4°C and supernatant was transferred to a new tube. Samples were stored at −80°C until used.

For SDS-PAGE, equivalent volumes of cell extract (5.6 µL of protein from exponential phase samples and 1.6 µL of protein from stationary phase samples) or culture supernatant (54 µL of 2X sample) were resolved by 12% or 10–20% SDS-PAGE (Protean II gel, Bio-Rad). Protein gels were stained with Gel-Code according to the manufacturer's instructions (Pierce). Images were adjusted for brightness and contrast in Adobe Photoshop CS (Adobe Systems Incorporated, San Jose, CA).

### Western blot for alpha-toxin and LukF-PV (PVL)

Overnight cultures of *S. aureus* were diluted 1∶100 in 5 ml of YCP medium and incubated at 37°C with shaking (250 rpm). Bacteria were cultured to mid-exponential (OD_600_ = 1.1) or stationary (OD_600_ = 1.7) phases of growth and removed by centrifugation (5000×g for 10 min at 4°C). Secreted proteins were precipitated using TCA (10% w/v), washed, and solubilized in 200 µl 10 mM Tris-Cl, pH 7.6. Proteins (15 µl of each sample) were resolved with 12% SDS-PAGE, followed by transfer to nitrocellulose. Membranes were blocked overnight at 4°C with 5% non-fat milk (Bio-Rad) and then incubated with rabbit polyclonal antibodies specific for LukF-PV (1 µg/ml, a kind gift of Dr. Gerard Lina, Lyon, France) or alpha-toxin (1∶10,000 dilution, Sigma-Aldrich, St Louis, MO) for 2 h at room temperature. Immunoblots were washed and incubated with horseradish peroxidase-coupled goat anti-rabbit IgG (1∶4,000 dilution, Zymed, CA) for 1 h at room temperature. Proteins were detected with enhanced chemiluminescence (GE, Piscataway, NJ) systems and autorad film (IscBioExpress, UT). Protein A (Spa) was detected by rabbit polyclonal antibody specific for Spa.

### Rabbit bacteremia model

Bacterial strains were grown in TSB at 37°C with shaking for 16–18 hours, harvested, and resuspended in 10% glycerol/phosphate buffered saline to approximately 5×10^8^ colony forming units (CFUs) per ml, aliquoted into individual cryovials and immediately stored at −80°C. For competition experiments, a mixture containing approximately 3×10^7^ CFUs of wild type parent and 3×10^7^ CFUs of isogenic Δ*pvl* mutant were used to co-infect New Zealand white rabbits via the marginal ear vein. A 0.5 ml volume of blood was sampled daily from the ear artery for quantitative blood culture. Rabbits were monitored twice daily to identify moribund animals (defined as those that are immobile, cannot be aroused to move from a recumbent position, and unable to access food or water) and those that have lost more than 15% of baseline weight, which are conditions stipulated by the UCSF committee on animal research for immediate euthanization. Rabbits were dead or euthanized for moribund conditions between 2 and 7 days post infection. A 0.2 to 0.3 g sample from lung, spleen and kidney was processed for quantitative bacterial culture onto blood agar plate (BAP; tryptic soy agar supplemented with 5% sheep blood; Remel, Lenexa, KS).

For single-strain experiments, 8×10^7^ CFUs of the parental strain SF8300 or the mutant strain SF8300Δ*pvl* were injected via the marginal ear vein into New Zealand white rabbits (no difference in CFUs of SF8300 or SF8300Δ*pvl* administered to rabbits, P>0.05 by unpaired Student's *t* test). Rabbits were euthanized and log_10_CFU per gram of lung, spleen, and kidney determined at 24, 48 and 72 hours post infection.

The animal experiments reported herein were reviewed by the University of California San Francisco Institutional Animal Care and Use Committee (IACUC). Animals were housed in humane conditions in accordance with IACUC policies and procedures.

### Statistics

For the rabbit co-infection model, the input ratio of the parent to mutant was determined by transferring 144 CFUs of the mixed inoculum onto TSA containing 300 µg/ml of spectinomycin. The parental strains were susceptible to spectinomycin, whereas the Δ*pvl* mutant strains were resistant because a spectinomycin resistance cassette was used in allelic replacement of wild type *pvl* genes [10]. Similarly, the output ratio of the parent and mutant was determined for each rabbit tissues (lung, spleen, kidney, and blood) by transferring 144 CFUs onto TSA containing 300 µg/ml spectinomycin. For each rabbit tissue, a competition index (CI) of the two comparator strains was calculated with the following formula that corrects the output ratios for variations in the input ratios: CI = log_10_(output ratio/input ratio). A positive CI value indicates enhanced tissue infectivity of the parent, whereas a negative CI value indicates enhanced tissue infectivity of the mutant; CI = 0 is the no-effect value. A paired Student's *t* test was used to test the null hypothesis (CI = 0) that there was no difference in tissue infectivity between the parent and the Δ*pvl* mutant. Linear trends in bacterial densities in vital organs and blood over time course of infection were explored by means of the Cuzick test (Stata 8, nptrend command) [31].
